# Population structure analyses and demographic history of the malaria vector *Anopheles albimanus *from the Caribbean and the Pacific regions of Colombia

**DOI:** 10.1186/1475-2875-8-259

**Published:** 2009-11-19

**Authors:** Lina A Gutiérrez, Nelson J Naranjo, Astrid V Cienfuegos, Carlos E Muskus, Shirley Luckhart, Jan E Conn, Margarita M Correa

**Affiliations:** 1 Grupo de Microbiología Molecular, Escuela de Microbiología, Universidad de Antioquia, Medellín, Colombia; 2 Programa de Estudio y Control de Enfermedades Tropicales-PECET, Sede de Investigación Universitaria-SIU, Universidad de Antioquia, Medellín, Colombia; 3 Department of Medical Microbiology and Immunology, University of California at Davis, USA; 4 Griffin Laboratory, Wadsworth Center, New York State Department of Health, Slingerlands, New York, USA

## Abstract

**Background:**

*Anopheles albimanus *is an important malaria vector in some areas throughout its distribution in the Caribbean and the Pacific regions of Colombia, covering three biogeographic zones of the neotropical region, Maracaibo, Magdalena and Chocó.

**Methods:**

This study was conducted to estimate intra-population genetic diversity, genetic differentiation and demographic history of *An. albimanus *populations because knowledge of vector population structure is a useful tool to guide malaria control programmes. Analyses were based on mtDNA *COI *gene sequences and four microsatellite loci of individuals collected in eight populations from the Caribbean and the Pacific regions of Colombia.

**Results:**

Two distinctive groups were consistently detected corresponding to *COI *haplotypes from each region. A star-shaped statistical parsimony network, significant and unimodal mismatch distribution, and significant negative neutrality tests together suggest a past demographic expansion or a selective sweep in *An. albimanus *from the Caribbean coast approximately 21,994 years ago during the late Pleistocene. Overall moderate to low genetic differentiation was observed between populations within each region. However, a significant level of differentiation among the populations closer to Buenaventura in the Pacific region was observed. The isolation by distance model best explained genetic differentiation among the Caribbean region localities: Los Achiotes, Santa Rosa de Lima and Moñitos, but it could not explain the genetic differentiation observed between Turbo (Magdalena providence), and the Pacific region localities (Nuquí, Buenaventura, Tumaco). The patterns of differentiation in the populations from the different biogeographic provinces could not be entirely attributed to isolation by distance.

**Conclusion:**

The data provide evidence for limited past gene flow between the Caribbean and the Pacific regions, as estimated by mtDNA sequences and current gene flow patterns among *An. albimanus *populations as measured by MS loci which may be mainly influenced by semi-permeable natural barriers in each biogeographical region that lead to the genetic differences and effective population sizes detected. The relatively high genetic differentiation in the port city of Buenaventura may be the result of specific ecological conditions, human migration and activities and/or differences in effective population sizes. This knowledge could serve to evaluate and coordinate vector control strategies in these regions of Colombia.

## Background

In Colombia, there are up to 100,000 malaria cases reported annually, approximately 10-20% of the cases in the Americas [1,2]. *Anopheles albimanus *is considered an important malaria vector in rural and periurban areas throughout its distribution in Colombia, along the Caribbean and Pacific Coasts and on San Andres Island [3]. A recent study showed that after 20 years it continues to be the predominant species in these coastal regions, accounting for 61 and 99% of the total capture of adult mosquitoes of the Caribbean and Pacific regions, respectively [4]. These regions represent substantial topographic, climatic and vegetation contrasts. In particular, the Caribbean coast is drier and has higher temperatures, while the Pacific coast is one of the rainiest regions globally, with high relative humidity and precipitation levels exceeding 5,000 mm/year [5]. The distribution of *An. albimanus *in Colombia is particularly remarkable because it includes coastal areas from three different biogeographic provinces, Maracaibo, Chocó and Magdalena in the neotropical Caribbean and Pacific regions of Colombia as described by Morrone [6].

The Pacific and the Caribbean regions are characterized by different levels of malaria transmission [1]. The Pacific region presents moderate to high transmission, while the Caribbean coast has presented low numbers of malaria cases and occasional epidemics [7]. *Anopheles albimanus *has been incriminated as a vector of *Plasmodium falciparum *and *Plasmodium vivax*, VK210 and VK247, in the Pacific region [4,8,9]. Despite its abundance in the Caribbean region and the high number of mosquitoes evaluated by Gutierrez *et al *[4], no infected individuals were detected, even though it was incriminated epidemiologically during a malaria outbreak in Guajira department in which *An. albimanus *was the predominant species [7]. This species is geographically variable in its infectivity by *Plasmodium *species throughout its distribution in the Americas [10-13].

Multiple factors could explain why *An. albimanus *has not been recently incriminated as a malaria vector in the Caribbean region: (i) because the number of human malaria cases reported from this region is lower than from the Pacific [1], more focused vector incrimination studies in this area are needed, (ii) Caribbean populations of *An. albimanus *could be more zoophilic because of extensive cattle ranching [3,5], (iii) different *Anopheles *species could be involved in malaria transmission at a local or regional level in the Caribbean coast [4,14,15], (iv) local adaptation between mosquito host and parasite could exist, as demonstrated in southern Mexico where reciprocal selection has led to local adaptation of *P. vivax*, and parasite populations are most compatible with their sympatric mosquito species (e.g., *An. albimanus *versus *Anopheles pseudopunctipennis*) [16], (v) the geographic, ecological and climatic differences between the regions could promote population differentiation of *An. albimanus *in Colombia and/or, (vi) demographic events could have influenced the current *An. albimanus *distribution and its role as a malaria vector in Colombia.

Interactions between changes in climatic conditions over large time scales with geographic features, sea level changes and contemporary factors such as human migration have influenced the distribution and diversification of different species [17-22]. For example, it has been suggested that the genetic differences detected in *Anopheles darlingi*, another important neotropical malaria vector, were affected by climate change at the end of the Pleistocene [23], Brazilian Amazon biogeography [24], and geographic barriers [25,26]. Similarly, studies of *An. albimanus *from Central and northern South America reported high variation in the intergenic spacers of nuclear ribosomal DNA [27] and differentiation between Central and South America using microsatellite loci and a mitochondrial DNA (mtDNA) marker [28,29]. Within Colombia, isoenzyme analyses revealed higher variability in Caribbean populations of *An. albimanus *than in Pacific populations and some loci showed significant allele frequency differences between these regions. However, cytogenetic data showed the same chromosomal banding patterns in all populations in the two regions, suggesting con-specificity in Colombia [30].

Several recent mtDNA studies have provided detailed accounts of species population structure and history [23,26,31-33], while studies based on highly polymorphic microsatellite loci have shown great potential for the evaluation of gene flow between populations of anophelines at a finer geographical and evolutionary scale [24,25,34-36]. Such studies have potential applied benefits, guiding and informing mosquito control strategies, such as the release of genetically modified mosquitoes refractory to the malaria parasite and the spread of insecticide resistance genes [37,38]. However, no mtDNA analyses or microsatellite loci information about malaria vector populations are available in Colombia. The taxonomic status of *An. albimanus *as a single lineage as defined by De Queiroz [39] is not disputed; however, additional detailed studies of inter-population genetic diversity could provide essential information about both historical and current distributions and processes affecting this species. Therefore, a focused population genetics and micro-evolutionary study of this important vector was conducted, using mtDNA cytochrome oxidase subunit I (*COI*) gene and four microsatellite markers, to address the hypothesis that historical and contemporary ecological and demographic processes may have led to some level of population differentiation of *An. albimanus *in the Caribbean and Pacific regions of Colombia. These data provide evidence for restricted past gene flow between the Caribbean and Pacific regions of Colombia as estimated by *COI *analysis, and contemporary gene flow among the regions as measured by four microsatellite markers (MS).

## Methods

### Sampling strategy

**Figure 1:**
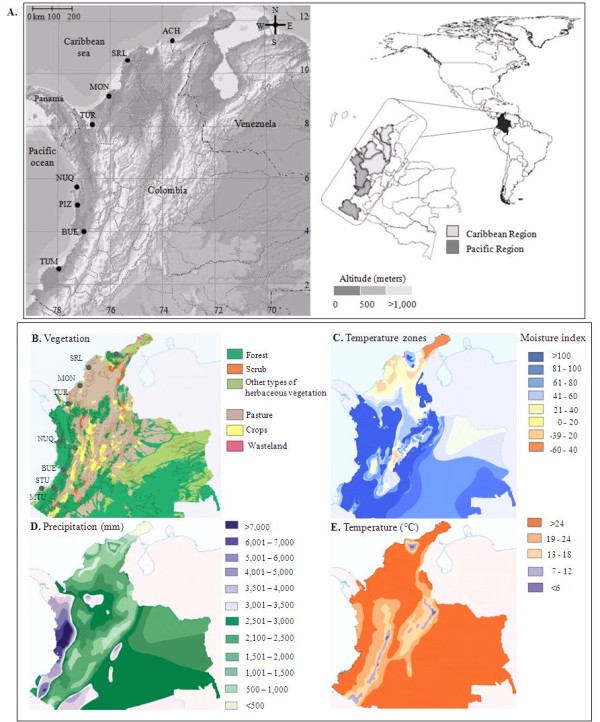
**A) Distribution of collection localities of *An. albimanus***. Altitude (meters) is based on a gray scale, and the distance scale is in km, **B) **Vegetation conditions of the Caribbean and the Pacific regions of Colombia, **C) **Temperature zones (according to Thornthwaite System) and moisture index, **D) **Average multi- annual precipitation (mm), **E) **Average multi- annual temperature (°C). Figures B to E were adapted from IGAC, with permission [5].

Adult *An. albimanus *specimens were collected from March 2005 to November 2007 in seven localities on the Caribbean and the Pacific coasts of Colombia (Figure 1, Table 1): Los Achiotes (ACH), Moñitos (MON), Santa Rosa de Lima (SRL), Nuquí (NUQ), Pizarro (PIZ), Buenaventura (BUE) and Tumaco (TUM). For details on sampling strategy and species identification see Gutiérrez *et al *[4]. Additional specimens collected in 2007 (Gutierrez *et al*, unpublished data) from Turbo (TUR), Antioquia, were also included (Figure 1, Table 1). Human landing catches of adults were conducted under an informed consent agreement using a protocol and collection procedures that were reviewed and approved by a University of Antioquia (SIU-UdeA) Institutional Review Board. ACH, SRL and MON are in the Maracaibo biogeographic province, NUQ, PIZ, BUE, and TUM are in the Chocó biogeographic province and TUR is in the Magdalena biogeographic province [6]. For *COI *sequencing samples from all sites were used, with individuals from Alto Guandipa in Mosquera (MTU) and La Ensenada in Santa Bárbara (STU), both sites in Tumaco, analysed as a single population (indicated as TUM; Figure 1A). For MS analysis, PIZ was excluded because there were not enough specimens with successful PCR amplification; STU and MTU from Tumaco were analysed separately because they are ~50 km apart (Figure 1B). Approximately 50% of the mosquitoes (randomly selected) included in this study were confirmed as *An. albimanus *using an ITS2-PCR-RFLP based assay [40,41].

**Table 1:** *Anopheles albimanus *collection data

Department	Locality (Abbreviation) Collection site*	Location (longitude/latitude)	Collection date (month/year)
**Caribbean Region**			
Magdalena	**Los Achiotes (ACH)**	11°15' N, 73°36' W	8/2005, 2, 6/2006
Bolívar	**Santa Rosa de Lima (SRL)**		7-10/2005, 2-3, 6/2006
	Cienaga	10°26' N, 75°21' W	
	Hatillo	10°25' N, 75°22' W	
Córdoba	**Moñitos (MON)**	9°15' N, 76°06' W	8-9/2005, 6-8, 11/2006
	Tierra Blanca	9°13' N, 76°08' W	
	Santander de la Cruz	9°11' N, 76°10' W	
Antioquia	**Turbo (TUR)**		11/2007
	Yarumal	8°07' N, 76°44' W	
	Camerun	8°08' N, 76°43' W	
			
**Pacific Region**			
Chocó	**Nuquí (NUQ)**	5°42' N, 77°16' W	3-6, 8-9, 11/2005, 2-3, 6/2006
	Panguí	5°39' N, 77°18' W	
	Panguí viejo	5°40' N, 77°17' W	
	**Pizarro (PIZ)**	4°57' N, 77°21' W	6/2005
Valle del Cauca	**Buenaventura (BUE)**		6, 10/2005, 2, 8/2006
	Barrio La unión	3°51' N, 77°0' W	
	La Barra	3°57' N, 77°22'W	
	Puerto España	4°02' N, 77°26' W	
Nariño	**Tumaco (TUM)**		12/2005, 10/2006
	Alto Guandipa	2°29' N, 78°26' W	
	La Ensenada	2°27' N, 77°58' W	

* For each locality the name of the collection site is given. *Collectors*: Naranjo, N.; Gutiérrez, L.; Cienfuegos, A.; Córdoba, L.; Solano, U.; Pinto, J.; Aviles, P.; Pozo, C.; García, L.; Quiñones, J. The specific collection sites are 2-11 km apart; two collection sites from TUM are ~50 km apart: Alto Guandipa, Mosquera (MTU) and La Ensenada, Santa Bárbara (STU).

### DNA extraction, *COI *gene amplification and sequencing

DNA was extracted from individual mosquito abdomens following a salt precipitation protocol [42]. The DNA was then resuspended in 25 μL of TE buffer (10 mM Tris, 1.0 mM EDTA) and stored at -20°C. A 1,300-bp segment of mtDNA *COI *gene was amplified by PCR using primers UEA3 5'-TAT AGC ATT CCC ACG AAT AAA TAA-3' and UEA10 5'-TCC AAT GCA CTA ATC TGC CAT ATT A-3' [43]. The PCR was performed in 25 μL containing 2 μL of DNA, 0.2 μM each primers, 1× reaction buffer, 1.5 mM MgCl_2_, 0.1 mM each dNTP, and 1 U Taq polymerase (Bioline, London, UK). The cycling conditions were: 5 min denaturation at 94°C, followed by 36 cycles of 1 min denaturation at 94°C, 1 min annealing at 50.2°C, and 1 min 15 seconds extension at 72°C, ending with a final extension at 72°C for 7 min. The PCR products were purified using the Wizard SV Gel and PCR Clean-Up System (Promega, Madison, WI), following the protocol recommended by the manufacturer. Both strands of the purified PCR products were sequenced, with a region of ~600-bp overlap, for 220 individuals. Only DNA sequence segments with higher than 30 Phred values [44,45] were used in the analyses. The forward and reverse chromatograms were manually corrected using the electropherogram viewer Chromas Lite^© ^[46]. Sequences from each mosquito were assembled by pairwise alignment using BioEdit Sequence Alignment Editor [47], then multiple alignment was performed using ClustalX [48]. Unique haplotypes were determined using DAMBE, version 4.5.68 [49]; identical sequences were considered to be a single haplotype.

### Microsatellite genotyping

The *An. albimanus *mosquitoes genotyped from each locality were selected randomly. Four di-nucleotide microsatellite (MS) loci described for *An. albimanus *by Molina-Cruz *et al *[29], were genotyped: 6-41, 1-90, 2-14 and 2-25. Each locus was amplified by PCR performed in a 25 μL volume containing 1 μL of DNA, 0.25 μM of each primer, 1× reaction buffer, 2.5 mM MgCl_2 _(6-41, 1-90, 2-14) or 2.0 mM MgCl_2_(2-25), 0.2 mM for each dNTP, and 1 U Taq polymerase (Bioline, London, UK). PCR additives were used as follows: 5% DMSO for reactions 1-90 and 2-25 and 0.5 μg/ml BSA for reaction 2-14. For all reactions, the cycling conditions were: 5 min denaturation at 95°C, followed by 35 cycles of 30 sec denaturation at 94°C, 20 sec annealing at 62°C (6-41), 50°C (1-90), 55°C (2-14), 58°C (2-25), and 30 sec extension at 72°C, ending with a final extension at 72°C for 10 min. Amplified fragments were separated by electrophoresis on DNA denaturing 6% polyacrylamide sequencing gels, and MS alleles were visualized by silver staining. To estimate allele sizes the length of bands was compared to a 10 bp DNA ladder (Invitrogen Inc., Carlsbad, CA, USA) over the migration/size of each MS allele using Quantity One^® ^software (Biorad Laboratories, Hercules, CA, USA). The most frequent homozygote MS alleles per locus were sequenced to provide reference sizes to estimate the number of repeats of other alleles. Allele sequences are available in GenBank [GenBank: FJ785408-FJ785419].

### Data analyses

#### Descriptive statistics

Indices of population and overall genetic diversity for *An. albimanus *were determined per locus using both haplotype frequencies of mtDNA *COI *gene and allelic frequencies of MS loci. Haplotype and nucleotide diversities were generated using DnaSP version 4.50.2 [50] and Arlequin version 3.11 software [51]. Nucleotide composition and patterns of nucleotide substitution were characterized using MEGA version 4.0 [52]. For the complete *COI *data set, an appropriate model of nucleotide substitutions was determined using the programme Modeltest 3.8 [53,54].

MICRO-CHECKER 2.2.3 software [55] was used to detect potential errors that may occur at each MS locus during genotyping or the interpretation of data such as null alleles, stuttering and large allele dropout. Allele and genotype frequencies of the amplified alleles were compared and adjusted if necessary for population genetic analysis. Number of alleles (*Na*), expected heterozygosity (*H*_*e*_), observed heterozygosity (*H*_*o*_), allele richness (*Rs*) and Hardy-Weinberg Equilibrium (HWE) were estimated for MS loci using GenAlEx version 6.1 [56] and FSTAT v 2.9.3.2 [57]. Statistical significance for HWE and linkage disequilibrium (LD) for each pair of loci was assessed using exact probability tests available in GENEPOP version 4.0 [58]. Whenever multiple comparisons were carried out simultaneously, the sequential Bonferroni procedure [59] was applied.

#### Population differentiation test

Using *COI *and MS data, genetic differentiation between populations was measured by estimating the fixation index *F*_ST_, using Arlequin software, and the significance was tested by permutation tests (10,000 replicates). Inbreeding coefficient (*F*_*IS*_) for each locus and overall loci by population [60] and the number of migrants per generation (*Nm*) between localities were also calculated in Arlequin. Effective population size (*Ne*) was estimated for each population under two methods, heterozygosity excess (HE) and linkage disequilibrium (LD) using NeEstimator software version 1.3 [61]. Geographical coordinates and distances of each sampling location were obtained using Google earth^® ^software [62], and the programme SAMOVA 12.02, Spatial Analysis of Molecular Variance, was used to maximize the proportion of total genetic variance due to differences between groups of populations as well as identifying possible genetic barriers between them, without prior information of the sampling locations as is necessary for AMOVA [63]. Analysis of Molecular Variance (AMOVA) was used to examine population variation among, within, and between collection sites. The probability that a sampled individual belonged to each reference population was estimated using assignment statistics in Geneclass 2.0 [64]. The Bayesian method of Rannala and Mountain [65] was selected as the computation criterion and the re-sampling algorithm; the method was performed with a minimum number of simulated individuals of 10,000 and a type I error of 0.01. In addition, the BAPS 5 programme (Bayesian Analysis of Population Structure) was used for Bayesian inference of genetic structure and admixture analyses performed for the *An. albimanus *populations [66]. A dendogram based on MS genetic distances was constructed using the Unweighted Pair Group Method Arithmetic average (UPGMA) cluster analysis in TFPGA programme version 1.3 [67] to test the genetic relationship among different populations. The correlation between genetic distance estimated from MS and geographical distances, assuming isolation by distance, was evaluated by the regression *F*_ST_/(1-*F*_ST_) on the natural logarithm (*Ln*) of pairwise geographical distances between collection sites. Significance of the correlation coefficient was assessed applying the Mantel test (10,000 randomizations) [68] using the Isolation By Distance Web Service version 3.15 [69].

#### Demographic inference and neutrality tests

DnaSP and Arlequin software were used to test the hypothesis that all mutations on *COI *gene are selectively neutral [70], employing Tajima's *D *[71] and Fu's *F*_*s *_[72]. Confidence intervals were tested by 10,000 coalescent simulations. Analyses for constant size and for growing populations were carried out to determine the distribution of the observed pairwise nucleotide site differences or mismatch distribution and the expected values (at equilibrium for no recombination) in a stable population or in growing and declining populations. Statistically significant differences between observed and model distributions were evaluated with the Sum of Squared deviation (SSD) and the raggedness statistic (*r*) [73] to reject the hypothesis of demographic expansion. Time since the population expansion can be estimated from *t *= *τ*/2*μ*, where *τ *(tau) is the date of the growth or decline measured in units of mutational time [*τ *= 2*μt*; *t *is the time in generations, and *μ *is the mutation rate per site (sequence size) and per generation] [74,75]. To analyse patterns of *An. albimanus *population history, haplotype networks were estimated by a parsimony-based method, which calculates the maximum number of mutational connections between pairs of sequences by the 95% parsimony criterion using the TCS computer software [76]. To obtain the most likely connection between two haplotypes and resolve some ambiguous loops in the network, several recommendations were followed [77-79]. Also, data were analysed using a neighbour-net network, which constructs split networks from inferred distance matrices, in the computer programme SplitsTree4, version 4.10 [80] and a neighbour-joining (NJ) algorithm was used to generate a tree in PAUP Version 4.0 [81] based on the genetic distance between haplotypes. For the latter method some analyses including an outgroup [82] were performed with *COI *gene sequences of *An. darlingi *collected from Colombia (unpublished data). The NJ tree and neighbour-net network analyses were performed with 1,000 bootstrap replications under the probability model identified using Modeltest. MS data were also used to estimate demographic processes such as recent population bottleneck and/or expansion, and heterozygosity tests were used to analyse deviations from Mutation-Drift Equilibrium (MDE) for each sample across all loci. At selectively neutral loci, the expected heterozygosity calculated from allele frequencies data (*He*) assuming HWE, and from the number of alleles and sample sizes (*Heq*), assuming a population at MDE, are expected not to be significantly different. Thus, if a significant number of loci show *He>Heq*, this indicates that the population recently experienced a bottleneck, conversely, a *He*<*Heq*, may suggest population expansion. Estimates of expected heterozygosity were calculated assuming Stepwise Mutation Model (SMM), Infinite Alleles Model (IAM) and Two Phase Model (TPM) with one-step mutation occurring at a frequency of 90% of the total. Statistical significance of the deviation from MDE was assessed by the sign test available in Bottleneck 1.2.02 [83].

## Results

### Intra-population genetic diversity

A 1,058-bp sequence of the *An. albimanus COI *gene corresponding to positions 1,802-2,859 of *Anopheles gambiae s.s*. mitochondrion complete genome and aligned with RefSeq NC 002084 [84] was analysed for 220 *An. albimanus *mosquitoes from eight localities (Figure 1A, Table 2). All 112 haplotype sequences are available in GenBank under the accession numbers: FJ015158-FJ015269. Sequences contained no missing data such as ambiguous base pairs and alignment revealed 107 variable sites, 67 of which were parsimony informative. A total of 115 nucleotide substitutions (with a majority predicted to introduce synonymous amino acid changes), 101 transitions and 14 transversions were observed. The best-fit DNA substitution model selected by the hierarchical likelihood ratio test (hLRT) was TrN+I+G [85], with invariable sites (*I *= 0.7675) and Gamma distribution shape parameter (*G*: 0.9555). Using Akaike Information Criterion (AIC) and Bayesian Information Criterion (BIC), the best-fit model was the Transitional model: TIM+I+G [86], with invariable sites (*I *= 0.7664) and Gamma distribution shape parameter (*G*: 0.8906), A-T composition of the *COI *sequences was 39.7% and 29.5%, respectively, similar to that found in other *Anopheles *species [23], and the above parameters were adjusted to construct the split networks from inferred distance matrices and the neighbour-joining analyses.

**Table 2:** Description of shared *COI *haplotypes and statistics of genetic polymorphisms for *An. albimanus*

Population	Haplotypes	*n*	*S*	*h*	*Hd *(SD)	*Pi *(SD)
ACH	A(3), C(9), F(2),	24	18	13	0.8551 +/- 0.065	0.0028 +/- 0.002
SRL	A(10), C(2), F(2), G(1), N(1),	30	26	19	0.8920 +/- 0.052	0.0031 +/- 0.002
MON	A(8), C(1), F(1), G(2), O(1), P(1)	29	27	20	0.9261 +/- 0.041	0.0030 +/- 0.002
TUR	A(6), C(1), G(1), H(2), M(1), N(1), O(1), P(1)	29	28	22	0.9581 +/- 0.028	0.0038 +/- 0.002
Caribbean Region	A(27), C(13), F(5), G(4), H(2), M(1), N(2), O(2), P(2)	112	58	61	0.9274 +/- 0.018	0.0035 +/- 0.002
						
NUQ	B(8), D(4), E(2), H(2), K(2), L(1)	30	50	15	0.9103 +/- 0.037	0.0103 +/- 0.005
PIZ	B(5), D(4), E(1), I(3), J(2), L(1)	24	42	14	0.9275 +/- 0.033	0.0097 +/- 0.005
BUE	B(11), E(1), I(1), J(1), M(1)	30	42	15	0.8483 +/- 0.056	0.0088 +/- 0.005
TUM	E(1), K(1),	24	33	19	0.9710 +/- 0.024	0.0078 +/- 0.004
Pacific Region	B(24), D(8), E(5), H(2), I(4), J(3), K(3), L(2), M(1)	108	72	53	0.9396 +/- 0.016	0.0098 +/- 0.005
						
All localities	-	220	107	112	0.9666 +/- 0.006	0.0152 +/- 0.007

*n*, number of mosquitoes sequenced; *S*, number of segregating sites; *h*, number of haplotypes; *Hd*, haplotype diversity; *Pi*, nucleotide diversity; (SD), Standard deviation. Number in parentheses represents the frequency of each haplotype by region or locality. Underlined haplotypes are shared between Caribbean and Pacific populations.

**Figure 2:**
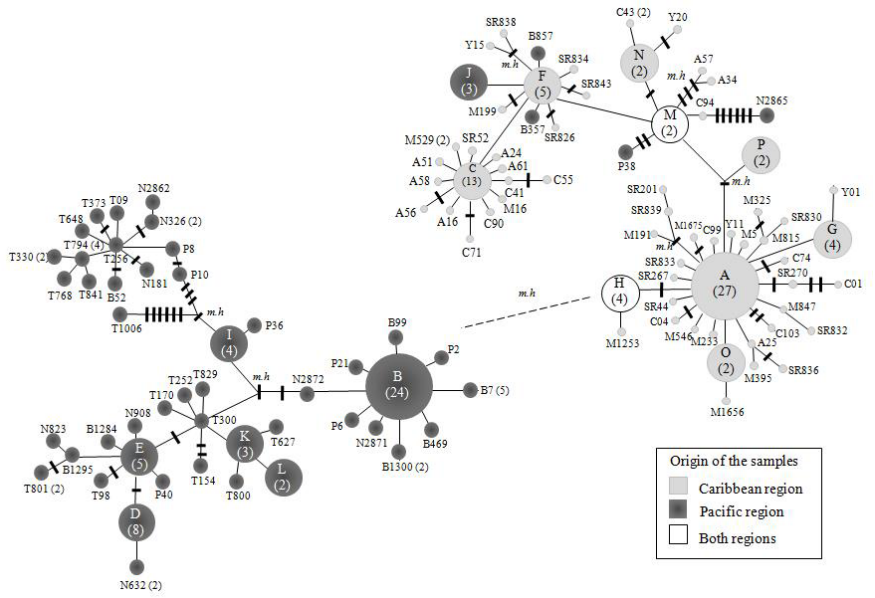
**Parsimony-based haplotype networks of 112 haplotypes from 1058 bp of the *COI *gene sequenced from 220 specimens of *An. albimanus***. The color of the circles and numbers in parentheses depict the origin and frequency of each haplotype, respectively. Each black bar represents one mutational step; the dashed line and *m.h *represent missing haplotypes. Unique haplotypes from Caribbean populations are represented in the network as Los Achiotes (A), Santa Rosa de Lima (SR), Moñitos (M) and Turbo (Y and C), and haplotypes from Pacific populations as Nuquí (N), Pizarro (P), Buenaventura (B) and Tumaco (T).

**Figure 3:**
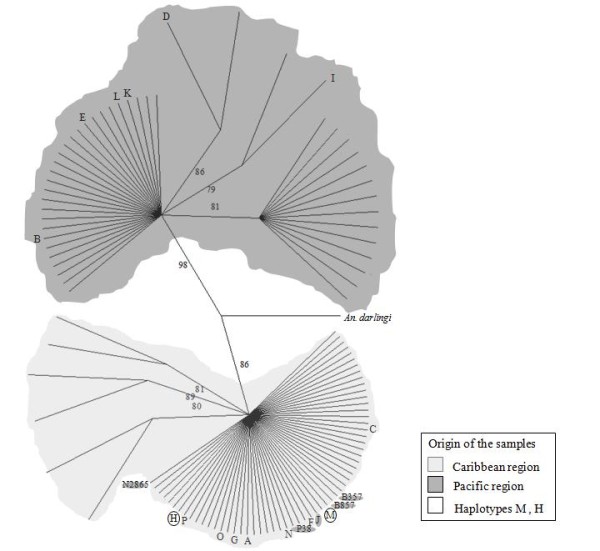
**Neighbour-Joining tree based on 112 haplotypes from 1058 bp of the *COI *gene sequenced from 220 specimens of *An. albimanus***. The percentage of trees in which the associated haplotypes clustered together in the bootstrap test (1,000 replicates) is shown next to the branches, retaining only groups with frequency ≥75%. Shared haplotypes (M, H) from the Caribbean and Pacific populations are represented in the tree.

Although diversity values were low overall, Pacific region populations had higher nucleotide diversities (0.0098, range 0.0078-0.0152) than Caribbean populations (0.0035, range 0.0028-0.0038), and haplotype diversity values were similar. There were 112 unique haplotypes (Table 2), 16 shared between different localities (named in order of frequency using capital letters), with the remainder exclusive to a particular geographic site (Figure 2, Table 2). In concordance with the parsimony-based network, the distance-based haplotypes networks (data not shown) and neighbour-joining tree (Figure 3) illustrated mainly two distinctive groups, corresponding to samples from the Caribbean or the Pacific regions.

For MS analyses, 280 *An. albimanus *were genotyped and the four microsatellite loci were polymorphic in all collection sites studied (Figure 1B). The number of alleles per locus ranged from three to twelve: locus 6-41 showed the lowest value and 1-90 the highest (Table 3). Allelic richness (*Rs*) per locus ranged between three in MON and MTU (6-41) to eleven, also, in MTU (2-25). STU showed the lowest average allelic richness (6.125), while BUE showed the highest (8.144) compared to all other localities. The expected heterozygosity (*He*) across all samples ranged from 0.503 (ACH) to 0.87 (MTU), while the average expected heterozygosity ranged from 0.681 (STU) to 0.784 (TUR). Of 32 tests performed for HWE, 12 (37.5%) remained significant after the sequential Bonferroni correction (*p *< 0.01); all of them were associated with heterozygote deficits, in each locus as follows: 6-41 (STU), 1-90 (TUR and BUE), 2-14 (ACH, MON, NUQ and BUE) and 2-25 (ACH, SRL, TUR, NUQ and BUE). Of 48 tests conducted, no locus was at linkage disequilibrium after the sequential Bonferroni correction (*p *< 0.05) (Table 3). The frequency of null alleles at each locus was compared; however, there were no significant changes in comparison with the initial results. Under LD the average *Ne *calculated for all populations was 308 individuals (CI 215-493) and the average *Ne *for all Caribbean populations (including TUR) was an infinite number of individuals (CI 1,613- ∞), while for all Pacific populations it was 256 individuals (CI 136- ∞). SRL showed the lowest value (231 individuals), and TUR, NUQ, STU and MTU showed an infinite result (∞). Under the HE model all of populations showed infinite values (See Additional file 1: Estimates of effective population size (*Ne*) and heterozygosity tests based on MS data for *An. albimanus*).

**Table 3:** Genetic variability estimates at MS loci in *An. albimanus *from three biogeographic provinces of Colombia

Locus		ACH	SRL	MON	TUR	NUQ	BUE	STU	MTU	All samples
		
		Maracaibo/Caribbean Region			Magdalena	Chocó/Pacific Region				
	**N**	40	40	40	40	40	40	21	19	280
	
**6-41**	***Na***	4	4	3	4	4	5	4	3	7
	***Rs***	3.473	3.475	3.000	3.475	3.921	4.334	3.898	3.000	3.675
	***H***_***e***_	0.503	0.617	0.627	0.662	0.583	0.623	0.484	0.555	0.659
	***H***_***o***_	0.325*	0.425*	0.625	0.575	0.525	0.450	0.381**	0.263*	0.446
	***F***_***IS***_	0.365	0.323	0.016	0.143	0.113	0.289	0.236	0.545	

**1-90**	***Na***	8	12	8	13	9	12	7	7	16
	***Rs***	7.075	9.702	7.681	10.63	8.077	9.526	6.904	7.000	8.866
	***H***_***e***_	0.742	0.838	0.813	0.848	0.805	0.819	0.746	0.742	0.813
	***H***_***o***_	0.650	0.800	0.7	0.800***	0.775	0.500***	0.714	0.632	0.683
	***F***_***IS***_	0.136	0.058	0.151	0.069	0.05	0.4	0.067	0.176	

**2-14**	***Na***	8	9	8	11	10	10	6	8	14
	***Rs***	7.321	7.886	6.132	8.469	8.512	8.902	5.898	8.000	8.606
	***H***_***e***_	0.811	0.807	0.674	0.792	0.802	0.830	0.717	0.794	0.812
	***H***_***o***_	0.700***	0.575*	0.600***	0.825	0.500***	0.525***	0.857	0.579	0.604
	***F***_***IS***_	0.149	0.299	0.122	-0.029	0.387	0.379	-0.173	0.295	

**2-25**	***Na***	10	9	10	9	9	11	8	11	12
	***Rs***	8.373	7.799	8.371	7.991	7.772	9.814	7.802	11.000	8.926
	***H***_***e***_	0.832	0.829	0.833	0.834	0.802	0.860	0.776	0.871	0.858
	***H***_***o***_	0.475***	0.600**	0.725	0.750**	0.375***	0.500***	0.571	0.789	0.558
	***F***_***IS***_	0.439	0.288	0.142	0.114	0.541	0.429	0.286	0.121	

**All *loci***	***Na***	7.5	8.5	7.25	9.25	8	9.5	6.25	7.25	7.94
	***Rs***	6.56	7.215	6.296	7.64	7.071	8.144	6.125	7.25	7.037
	***H***_***e***_	0.722	0.773	0.737	0.784	0.748	0.783	0.681	0.741	0.746
	***H***_***o***_	0.537	0.600	0.663	0.738	0.544	0.494	0.631	0.566	0.596

N: Number of mosquitoes, *Na*: number of alleles, *Rs*: Allele richness; *H*_*e*_: expected heterozygosity, *H*_*o*_: observed heterozygosity, *F*_*IS*_: inbreeding coefficient. All loci/samples: mean values over loci or collecting sites. Asterisks are showing significant heterozygote deficits according to exact tests against Hardy-Weinberg proportions after the sequential Bonferroni correction; *p < 0.05, **p < 0.01, ***p < 0.001.

### Genetic structure and differentiation

In the AMOVA analyses using haplotype frequencies with the eight populations separated into two groups, including TUR in the Caribbean region, 5.85% (*p *< 0.05) of the total variance was explained at the among regions level. When populations were separated into two groups, including TUR in the Pacific region, only 3.86% (*p *< 0.05) of the total variance was due to the variation among regions (Table 4).

**Table 4:** Analysis of Molecular Variance (AMOVA) using *COI *and MS data in *An. albimanus *from Colombia

Source of variation	Variance components	Percentage of variation	***F***_***ST***_
MS Two groups Among the Caribbean and the Pacific regions (including TUR in the Caribbean region)	0.04944	3.08	0.05040*
Among populations within regions	0.03140	1.96	
Within populations	1.52313	94.96	

MS Two groups Among the Caribbean and the Pacific regions (including TUR in the Pacific region)	0.05419	3.37	0.05162*
Among populations within regions	0.02871	1.79	
Within populations	1.52313	94.84	

*COI *Two groups Among the Caribbean and the Pacific regions (including TUR in the Caribbean region)	0.02925	5.85	0.08934*
Among populations within regions	0.01539	3.08	
Within populations	0.45509	91.07	

*COI *Two groups Among the Caribbean and the Pacific regions (including TUR in the Pacific region)	0.01916	3.86	0.08262*
Among populations within regions	0.02183	4.40	
Within populations	0.45509	91.74	

* *p *< 0.05; significant level based on 1,023 permutations

The *F*_*ST *_value estimated from *COI *sequences for the comparison between the Caribbean and Pacific regions was 0.07 (*p *< 0.05), and most of the pairwise comparisons of *F*_*ST *_between localities were significant; however, no significant *F*_*ST *_values at *p *< 0.01 were observed in comparisons between SRL, MON and TUR from the Caribbean coast, or between NUQ, PIZ and BUE from the Pacific coast (Table 5). Estimates of pairwise genetic differentiation (*F*_*ST*_) and gene flow (*Nm*) among populations using MS data are shown in Table 6. Overall significant *F*_*ST *_values were observed between population belonging to the same region, Caribbean or Pacific. Of 28 differentiation tests, 18 were significant (*p *< 0.01). Comparisons between ACH and STU showed the highest degree of differentiation. *An. albimanus *samples collected from TUR, located in the middle of the collection range, showed lower differentiation with its nearest localities: NUQ (Pacific) and MON and SRL (Caribbean). *Nm *estimates among *An. albimanus *populations ranged from 4 to 59 individuals, presenting an average value of 21 individuals.

**Table 5:** *F*-Statistics based on pairwise estimates of *COI *haplotype frequencies of *An. albimanus *from 8 localities in Colombia

Populations		ACH	SRL	MON	TUR	NUQ	PIZ	BUE	TUM
**ACH**		___							
**SRL**		0.05820**	___						
**MON**		0.06177**	-0.00873	___					
**TUR**		0.05614**	0.00155	-0.00548	___				
**NUQ**		0.11671**	0.09885**	0.08179**	0.06150**	___			
**PIZ**		0.10870**	0.09058**	0.07319**	0.05698**	-0.00089	___		
**BUE**		0.14841**	0.12989**	0.11293**	0.09591**	0.02299	0.03041*	___	
**TUM**		0.08696**	0.06914**	0.05171**	0.03550**	0.05450**	0.04907**	0.09020**	___

**p < 0.01; * *p *< 0.05

**Table 6:** Estimates of pairwise genetic differentiation (*F*_*ST*_) and gene flow [48] among populations of *An. albimanus *based on MS data

Populations	ACH	SRL	MON	TUR	NUQ	BUE	STU	MTU
**ACH**	__	59	31	13	6	9	4	7
**SRL**	0.00834	__	56	38	8	11	5	10
**MON**	0.01602*	0.00889	__	26	10	15	5	11
**TUR**	0.03596***	0.01286*	0.01880**	__	47	22	12	24
**NUQ**	0.08121***	0.05584***	0.04891***	0.01046	__	18	41	35
**BUE**	0.05404***	0.04186***	0.03227***	0.02211**	0.02697**	__	10	35
**STU**	0.11405***	0.09112***	0.09023***	0.04015***	0.01188	0.04721***	**__**	15
**MTU**	0.06623***	0.04589**	0.04233***	0.02050*	0.01439	0.01421	0.03274*	**__**

Above diagonal: *N*_*m *_values; below diagonal: *F*_*ST *_values; * *p *≤ 0.05; ** *p *≤ 0.01; *** *p *≤ 0.001

**Figure 4:**
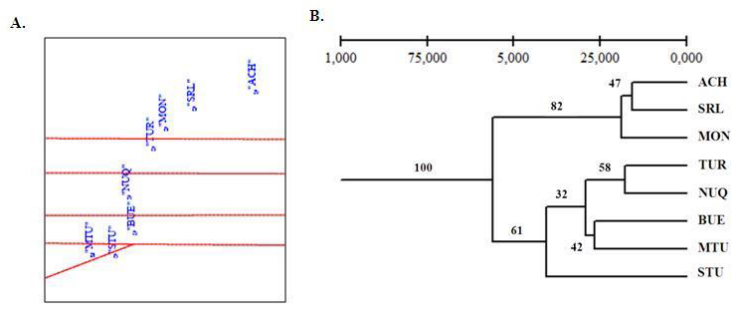
**Cluster analysis based on MS data**. **A**: Map of the sampling points and the barriers between the groups of populations defined by SAMOVA. **B**: UPGMA dendogram based on Nei's genetic distance for the eight populations of *An. albimanus*.

The SAMOVA was used to estimate heterogeneity among populations from the Caribbean and Pacific regions. This test maximized the proportion of total genetic variance between populations at six groups: 1) ACH, SRL and MON, 2) TUR, 3) NUQ, 4) BUE, 5) STU, 6) MTU (Figure 4A). In agreement with SAMOVA data, the UPGMA dendogram based on genetic distances showed two distinctive groups in which ACH, SRL and MON constituted a cluster (82% support) and TUR, NUQ, BUE, STU and MTU were included in a second one at 61% support (Figure 4B). Given the number of MS loci tested in this study and in the low support values for the UPGMA dendogram, the possibility that data on the apparent relationship between populations from the Caribbean and Pacific regions may be biased cannot be excluded. However, BAPS clustering was also congruent with the results obtained by SAMOVA and UPGMA analyses, which proposed two groups without admixture among them, except for TUR which showed signs of admixture with both. AMOVA was conducted with both the *COI *and MS data to test the Isthmus of Panama as a putative barrier between the Caribbean and Pacific regions, including and excluding TUR. The AMOVA corresponding to two groups: the Caribbean and the Pacific samples (excluding TUR), showed the highest percentage of the total variance and *F*_*ST *_value, 4.3% (*p *< 0.05) and 0.06049 (*p *< 0.05), respectively (Table 4). Results of the assignment statistics showed that on average 28.6% (80 of 280) of the individuals were correctly assigned to their original reference site. Samples from STU and BUE presented the highest proportion of correctly assigned individuals (38%). In general, mis-assignments occurred between samples of the same region, either the Caribbean or the Pacific (Table 7).

**Table 7:** Data of assignment tests based on MS among samples of *An. albimanus *from Colombia

Populations	ACH	SRL	MON	TUR	NUQ	BUE	STU	MTU
ACH	0.25*	0.20	0.18	0.08	0.05	0.08	0.03	0.15
SRL	0.20	0.20*	0.20	0.13	0.10	0.05	-	0.13
MON	0.18	0.10	0.35*	0.05	0.13	0.10	0.03	0.08
TUR	0.03	0.18	0.10	0.33*	0.18	0.05	0.08	0.08
NUQ	0.05	0.03	0.10	0.23	0.25*	0.05	0.15	0.15
BUE	0.10	0.03	0.08	0.05	0.10	0.38*	0.15	0.13
STU	-	-	-	-	0.33	0.10	0.38*	0.19
MTU	0.11	0.16	0.11	-	0.11	0.05	0.37	0.11*

Values are proportions of mosquitoes from the original reference site (rows) assigned to each population site (columns), *Proportions of samples correctly assigned.

A correlation test between genetic distance based on MS loci, measured by *F*_ST_/(1- *F*_ST_), and the geographic distance (Ln) was statistically significant, although with low values, for the whole dataset (Mantel tests: *R*^*2 *^= 0.36, *p *= 0.003). The IBD model best explains genetic differentiation among populations from the Caribbean region: ACH, SRL and MON (excluding TUR), but it could not explain the low differentiation observed within the Pacific region populations (NUQ, BUE, STU and MTU). No statistically significant correlation was detected when tests of IBD were carried out separately for the Caribbean (including TUR) or the Pacific region (with or without TUR).

### Demographic inference and neutrality tests

The most common haplotypes were A (*n *= 27), exclusively Caribbean, and B (*n *= 24), exclusively Pacific. Three localities (TUR, NUQ and BUE) share haplotypes H and M from both regions (Figure 2, Table 2). Haplotypes from the two regions differed by more than 13 mutational steps. Interestingly, the shared haplotypes (H, M) and the Pacific haplotypes J, P38, B357, B857 and N2865 clustered with the Caribbean region (Figure 2). Haplotypes A and B were the most common interior haplotypes and so are most likely to be ancestral [87]. The majority of haplotypes in both regions were tip alleles, considered to be more recently derived and geographically restricted [77,87]. In addition, private haplotypes located peripherally are suggestive of a demographic expansion [72,75] within the Caribbean region with subsequent limited gene flow between the two regions. In general, the parsimony-based network (Figure 2), distance-based haplotype networks and neighbour-joining tree (Figure 3) consistently show two distinctive groups, with a large star-shaped Caribbean network surrounding haplotype A, a smaller network around haplotype C, and a small Pacific network surrounding haplotype B. In the NJ tree, the Caribbean haplotypes were supported at 86% and the Pacific haplotypes at 98% (Figure 3), and the bootstrap support level was 99.9% for the branch that connects Caribbean and Pacific haplotypes in the *An. albimanus *neighbour-net network.

Both neutrality tests found the Caribbean populations have significant negative values, and one test was significant for the Pacific populations (see additional file 2: Results of neutrality tests based on *COI *sequences of *An. albimanus *from Colombia), which suggested a possible past demographic expansion event. The distribution of the number of differences (mismatches) between pairs of DNA sequences from Caribbean populations demonstrated the expected unimodal distribution for Caribbean all populations together and also for each population. The expected distribution did not differ significantly from the sudden-expansion model. The distribution for each locality from the Pacific region and for the grouped Pacific region showed a multimodal distribution typical of populations at equilibrium. Although the *r *(0.009; *p *= 0.91) and SDD (0.002; *p *= 0.75) values were not significant for Caribbean populations, the estimated values were small, also supporting a population expansion. As in previous *Anopheles *studies [23,31], the *Drosophila *(*D. melanogaster *and *D. yakuba*) mutation rate values of 10^-8^/site/year [88] and 10 generations/year [89] were assumed to estimate the time for the Caribbean population expansion. For *An. albimanus*, using *τ *= 4.654, this is approximately 21,994 years ago (95% CI, 8,969-34,347) during the late Pleistocene.

The heterozygote test performed under SMM, IAM and TPM using MS data showed different results. Using IAM, heterozygote excess was detected for all of the populations tested; however, none were statistically significant. Under SMM four populations showed heterozygote deficits: STU, BUE, NUQ and ACH; only STU was statistically significant. For TPM three populations presented heterozygote excess: MON, SRL and NUQ; only NUQ was statistically significant. STU showed heterozygote deficits that were not statistically significant.

## Discussion

### Distinctive evolutionary genetics and demographic history evidence

Nucleotide diversity values for *An. albimanus COI *sequences, including synonymous and non-synonymous changes, were similar to those found for *An. darlingi COI *sequences from Central and South America [23], *An. gambiae *[84] and other species within the Insecta class. In particular, all are characterized by having an adenine-thymine rich mitochondrial genome. *Anopheles albimanus COI *sequences presented a combined frequency of ~70% AT. The results show that *An. albimanus *populations from the Caribbean and Pacific regions in Colombia have moderately high genetic diversity, in contrast to the lower diversity in Pacific localities detected using isozymes [30]. This difference is likely a result of the greater sensitivity of DNA markers to detect genetic variability, since DNA polymorphic sites are not necessarily seen at the protein level [90]. Nucleotide diversity was higher in most of the Pacific populations; this may indicate that different *An. albimanus *maternal lineages invaded these two Colombian regions at different times and (or) that the Pacific populations are older relative to the Caribbean ones, perhaps most closely related to populations from Cuba and Central America, which had similar values using *ND5 *sequence [29].

Panmictic populations of *An. albimanus *(within ~665 km) had been observed in Central America, Costa Rica and Panama, Colombia and Venezuela. It appears that *An. albimanus *populations have had different periods of isolation followed by secondary contact throughout the species range in America [27-29]. The data showed some haplotypes from Pacific populations clustering with the Caribbean group (Figure 2), possibly a genetic signature of a panmictic gene pool that existed before the late Pleistocene. Multiple factors may be responsible for the observed distribution and frequency of haplotypes from the Caribbean and Pacific regions in Colombia. Most haplotypes are not shared between regions, perhaps because of the distance between populations (>200 km), distinctive demographic history, human impact (insecticide use could have led to local haplotype extinctions) and/or distinctive ecological conditions in each region. Previous mtDNA analysis of *An. albimanus *did not detect a significant correlation between genetic and geographic distance [29]. Although, genetic differences and geographical distances were not directly compared because the populations were not in MDE, distance did not appear to be a significant factor affecting the data.

The neutral model is rejected for the Caribbean populations, indicating possible previous population expansion and/or natural selection. In general, all tests illustrate two distinctive groups, corresponding to haplotypes from the Caribbean and from the Pacific regions. The data did not show significant differences between *An. albimanus *populations from TUR, located in the Magdalena province as described by Morrone [6], and the other populations from the Caribbean coast. The relatively strong mtDNA support for expansion in the Caribbean *An. albimanus *populations may indicate different demographic histories for this species in these two regions of Colombia, and the AMOVA support for variance resulting from these two groups is significant, albeit low. Population expansion for *An. albimanus *from the Caribbean coast in Colombia is estimated to the late Pleistocene, similar to *An. darlingi *[23].

### Contemporary population structure

Microsatellite loci used in this study have not been physically mapped in *An. albimanus *polytene chromosomes, consequently, their location on chromosome inversions is unknown and neutrality cannot be assumed [91]. Chromosomal inversions have not been detected in *An. albimanus *and it is conspecific along its distribution [30,92]; however, the microsatellite loci analysed in this study were highly polymorphic (Table 3), showing their usefulness for evaluating the population structure of *An. albimanus *in Colombia.

Significant departures from HW equilibrium were detected associated with heterozygote deficits (Table 3), similar to that reported in other anopheline microsatellite studies [25,29,34,93,94]. Heterozygote deficits are attributed to either significant inbreeding, Wahlund effect, natural selection or the presence of null alleles [24,95,96]. Inbreeding as the possible cause of heterozygote deficits is not considered due to the fact that it affects all loci equally, which is not compatible with the heterogeneity detected in this study (Table 3). The Wahlund effect refers to reduction of heterozygosity in a population caused by subpopulation structure [97]. In the data, SAMOVA, UPGMA and BAPS based on MS loci identified some degree of population subdivision, mainly among the Caribbean and the Pacific regions, and within the Pacific region, thus a part of the heterozygote deficits detected could be the result of Wahlund effect. High levels of heterozygote deficits could also be the result of null alleles, as a result of accumulation of mutations in the primer binding sites [34]. In this study, there was failure of amplification of different loci for some specimens; however poor DNA quality was discarded as a cause, similarly, Molina-Cruz *et al *[29], observed similar cases of amplification failure. In summary the most likely causes of heterozygote deficits in the present study were null alleles and Wahlund effect.

The moderate level of population differentiation detected (*F*_*ST*_) was not observed with isozyme markers [30]. In theory, the analysis of a low number of loci could potentially increase population differentiation values (*F*_*ST*_), but at the same time, the *F*_*ST *_is the estimate presenting the lowest differentiation bias [98]. The SAMOVA, UPGMA and BAPS detected two distinctive clusters: ACH, SRL, MON and TUR, NUQ, BUE, STU and MTU. Unlike the *COI *data, the TUR population clustered with the Pacific region populations. Low admixture was detected between the cluster represented by ACH, SRL and MON from the Caribbean region and the other five populations. Thus, the largest genetic differentiation was observed in comparisons among the Caribbean and the Pacific region, and SAMOVA analyses placed TUR, NUQ, BUE, STU and MTU in five different groups perhaps suggesting a specific barrier that reduces gene flow between these populations.

Data could suggest that features related to the three different biogeographic provinces, Maracaibo, Magdalena and Chocó [6], such as vegetation, weather, moisture, precipitation and temperature (Figure 1B-E) [5], constitute a semi-permeable barrier that reduces gene flow of *An. albimanus *populations at the inter-regional or the inter-biogeographic provinces level. Also the differences in *N*_*e *_detected among the populations may have contributed to the genetic differentiation of these regions [99]. Because some differentiation was observed between populations in the Chocó province (Pacific region) it is possible that other processes besides ecological conditions may affect free gene flow between *An. albimanus *populations.

The level of differentiation (*F*_*ST*_), among the Caribbean and the Pacific region of Colombia is comparable to that reported among countries in South America and between Cuba and continental *An. albimanus *populations (*F*_*ST *_= 0.057 and 0.059, respectively) [29]. Similar levels of differentiation were also found among *Anopheles nuneztovari *s.l from Cordoba, Norte de Santander and Valle in the east, west and southeast of Colombia (*F*_*ST *_= 0.024-0.06), using RAPDs (Randomly Amplified Polymorphic DNA) [100]. In this study, the authors suggested IBD could explain their results. In a study of *An. darlingi *populations from Cordoba, Meta and Chocó in the northwest and west of Colombia, using AFLPs (Amplified Fragment Length Polymorphism) and RAPDs (RAPD *F*_*ST *_= 0.084, AFLP *F*_*ST *_= 0.229), results suggested that the observed genetic differences could be the result of the biogeographic characteristics of each particular region [101]. The data showed that in addition to the differences in the demographic history of each region, the presence of semi-permeable biogeographic barriers, could contribute to the differentiation observed using MS.

In general, in the Caribbean region, there was low genetic differentiation, partly explained by the high *Ne*, that could have increased gene flow and decreased population genetic structure [24]. IBD was the model that best explained differentiation among ACH, SRL and MON; however, if TUR was included in the analysis, the resulting data did not fit this model. TUR, the most genetically distant Caribbean region population, (Figure 4B), could be influenced by its location in Magdalena biogeographic province [6], and by differences in the effective population sizes. Similarly, low non-significant differentiation was observed among the Pacific region populations between NUQ-STU (362 Km), NUQ-MTU (371 Km) and BUE-MTU (217 Km) (Table 5) and the level of differentiation observed was not congruent with IBD.

A significant level of differentiation among the populations closer to BUE was detected, probably influenced by ecological characteristics [5] that could reduce gene flow with NUQ and STU (Figure 1B-E), in addition to their differences in effective population sizes. Ecologic and climatic variation with appropriate conditions for mosquito development have been recognized among the main causes for peaks of mosquito abundance and subsequent peaks of malaria cases [102]. Therefore, further studies would be essential in BUE in comparison with others localities from the Pacific region. Even though there was restricted gene flow with respect to adjacent populations, BUE presented the highest genetic diversity, characterized by a high proportion of private alleles in low frequency. This could be explained by the characteristics of BUE, the main Colombian port city, where human migration and activities may promote gene flow among mosquito populations with different genetic pools, thereby increasing variability, as has been reported for *An. gambiae *[35,103,104]. The levels of differentiation observed between MTU and STU could also be due to ecological differences [5]; however, an overestimation of differentiation (*F*_*ST*_), is not discarded given the low sample number [97,105].

Most population structure analyses assume equilibrium, however, in several studies on anopheline vectors this has been violated, for example for *An. gambiae *and *Anopheles arabiensis *[106], *Anopheles dirus *[94], *Anopheles moucheti *[36] and *An. darlingi *[34]. In this study, the heterozygosity test did not detect a significant departure from equilibrium for most of the populations. Nevertheless, under the TPM model, there was evidence of a bottleneck for NUQ which was not sustained by the SMM and IAM models and in disagreement with the effective population size observed, perhaps because of the mutational model and low number of analysed loci [83]. Under the SMM model, the STU population appears to be expanding, consistent with the effective population size.

The *Ne *calculated under the HE model assumes equilibrium [107]. Some of the Caribbean and Pacific populations were at H-W disequilibrium for some loci; it is possible that the departure from equilibrium influenced the values detected with HE, nevertheless, the confidence intervals obtained with LD agreed with those of HE. In general, high *Ne *values were obtained for all localities and on average, the effective population size (*Ne *= 308 individuals) was three fold higher than in the study with *An. albimanus *from Central and South America (*Ne *= 96 individuals) [29]. For TUR and the Pacific region populations, except BUE, an infinite *Ne *was obtained under both models. According to Donnelly *et al*. [99] heterogeneity in the *Ne *could contribute to the levels of population genetic differentiation seen in this study. Similarly, *Ne *heterogeneity has been reported for *An. darlingi *[24,34] and within the *An. gambiae *complex [108-111]. In *An. gambiae*, the *Ne *heterogeneity observed in the savana cytotype was demonstrated to be region specific [111]. In the present study, high *Ne *might be associated with a wide availability of breeding places that are suitable for *An. albimanus*, such as lakes, ponds, mine excavations and fish ponds [3,112]. The high precipitation levels characteristic of the Pacific region [5], could contribute significantly to the availability of a variety of breeding sites for *An. albimanus*.

## Conclusion

The genetic diversity of Caribbean and Pacific populations of *An. albimanus *detected using *COI *gene sequences appears to be mostly influenced by demographic events in *An. albimanus *populations during the late Pleistocene. The data indicated little to moderate genetic differentiation among *An. albimanus *populations from the Caribbean and the Pacific regions. Current gene flow patterns may be mainly influenced by semi-permeable natural barriers in each biogeographical region that lead to the genetic differences and effective population sizes found in this study.

Current hydrological and climatic variation in Colombia is a factor associated with malaria outbreaks for multiple localities throughout endemic regions of the country [113]. Therefore additional studies could test the influence of specific ecological and climatic conditions on the genetic differentiation in *An. albimanus *populations from the Caribbean and Pacific regions. It would also be of interest to examine additional populations of other *Anopheles *species to evaluate the generality of these patterns and to test the hypothesis that Caribbean expansion occurs among the *Anopheles *species whose distributions include these Colombian geographic areas.

In summary, this study showed current patterns of gene flow among the different *An. albimanus *populations from Colombia. This knowledge could be applied to existing vector control strategies since data on vector population genetics allow the inference of the spread of genes important for insecticide resistance or refractoriness to malaria parasites. In addition, this information contributes to the understanding of the epidemiology and the dynamics of disease transmission [99]. Moreover, effective population sizes calculated in this study may be used to evaluate local control measure effectiveness.

## Competing interests

The authors declare that they have no competing interests.

## Authors' contributions

LAG contributed in the design of the study, extracted DNA, performed all *COI *molecular procedures and analysis, co-participated in MS genotyping and analysis and drafted the manuscript. NJN contributed in the design of the study, was involved in field surveys, conducted procedures for microsatellite genotyping and analysis and helped in manuscript preparation. AVC co-participated in the design of the study and performed all molecular identification assays. CEM, SL and JEC co-participated in the design of the study and helped drafting the manuscript. MMC participated in the design of the study, data analysis, draft of the manuscript, general supervision of the research group and funding acquisition. All authors read and approved the final document.

## Supplementary Material

Additional file 1 **Estimates of effective population size (*Ne*) and heterozygosity tests based on MS data for *An. albimanus***. The table provided include the estimated values of effective population size (*Ne*) and heterozygosity tests based on MS data for *An. albimanus *from eight sites from the Caribbean and the Pacific regions of Colombia. Click here for file

Additional file 2 **Results of neutrality tests based on *COI *sequences of *An. albimanus *from Colombia**. The table provided describe the estimated values of neutrality tests based on *COI *sequences of *An. albimanus *from eight sites from the Caribbean and the Pacific regions of Colombia. Click here for file

## References

[B1] INS Estadísticas del Sistema de Vigilancia en Salud Pública - SIVIGILA, Casos Totales en la Semana Epidemiológica 52 y Acumulados del Año 2007 Boletín Epidemiológico Semanal. Instituto Nacional de Salud, Subdirección de Vigilancia y Control en Salud Pública de Colombia, Bogotá

[B2] PAHO Regional Strategic Plan for Malaria in the Americas 2006 - 2010 2006 PAHO HQ Library Cataloging-in-Publication Data. Pan American Health Organization, Washington, D.C

[B3] Olano VA Brochero H Sáenz R Quiñones M Molina J Mapas preliminares de la distribución de especies de *Anopheles *vectores de malaria en Colombia Biomedica 2001 21 402 408

[B4] Gutiérrez LA Naranjo N Jaramillo LM Muskus C Luckhart S Conn JE Correa MM Natural infectivity of *Anopheles *species from the Pacific and Atlantic Regions of Colombia Acta Trop 2008 107 99 105 10.1016/j.actatropica.2008.04.019 18554564

[B5] IGAC Atlas de Colombia CD-ROM 2002 5a Bogotá DC, Colombia: Instituto Geográfico Agustín Codazzi

[B6] Morrone JJ Biogeographic areas and transition zones of Latin America and the Caribbean islands based on panbiogeographic and cladistic analyses of the entomofauna Annu Rev Entomol 2006 51 467 494 10.1146/annurev.ento.50.071803.130447 16332220

[B7] Cáceres DC De La hoz F Nicholls S DeAntonio R Velandia MP Olano VA Montoya R Pinzón E García M Flórez AC Bruzón L Burbano ME Bonivento J Brote de malaria en La Guajira, 1 de diciembre de 1999 a 1 de febrero de 2000 Biomedica 2000 20 152 161

[B8] Herrera S Suarez M Sanchez G Quiñones M Herrera M Uso de la técnica inmuno-radiométrica (IRMA) en *Anopheles *de Colombia para la identificación de esporozoitos de *Plasmodium* Colomb Med 1987 18 2 6

[B9] Quiñones M Suárez M Fleming GA Distribución y bionomía de los anofelinos de la Costa Pacífica de Colombia Colomb Med 1987 18 19 23

[B10] Gonzalez-Ceron L Rodriguez MH Santillan FV Hernandez JE Wirtz RA Susceptibility of three laboratory strains of *Anopheles albimanus *(Diptera: Culicidae) to coindigenous *Plasmodium vivax *circumsporozoite protein phenotypes in southern Mexico J Med Entomol 2000 37 331 334 10.1603/0022-2585(2000)037[0331:SOTLSO]2.0.CO;2 15535573

[B11] Olano VA Carrillo MP Espinal CA Estudios de infectividad de la especie *Anopheles albimanus *Wiedemann 1820 (Díptera: Culicidae) cepa Cartagena con plasmodios humanos Biomedica 1985 5 5 10

[B12] Rodriguez MH Gonzalez-Ceron L Hernandez JE Nettel JA Villarreal C Kain KC Wirtz RA Different prevalences of *Plasmodium vivax *phenotypes VK210 and VK247 associated with the distribution of *Anopheles albimanus *and *Anopheles pseudopunctipennis *in Mexico Am J Trop Med Hyg 2000 62 122 127 10761736 10.4269/ajtmh.2000.62.122

[B13] Warren M Collins WE Richardson BB Skinner JC Morphologic variants of *Anopheles albimanus *and susceptibility to *Plasmodium vivax *and *P. falciparum* Am J Trop Med Hyg 1977 26 607 611 329696 10.4269/ajtmh.1977.26.607

[B14] Póvoa MM de Souza RT Lacerda RN Rosa ES Galiza D de Souza JR Wirtz RA Schlichting CD Conn JE The importance of *Anopheles albitarsis *E and *An. darlingi *in human malaria transmission in Boa Vista, state of Roraima, Brazil Mem Inst Oswaldo Cruz 2006 101 163 168 10.1590/S0074-02762006000200008 16830709

[B15] Quiñones ML Ruiz F Calle DA Harbach RE Erazo HF Linton YM Incrimination of *Anopheles *(*Nyssorhynchus*) *rangeli *and *An*. (*Nys*.) *oswaldoi *as natural vectors of *Plasmodium vivax *in Southern Colombia Mem Inst Oswaldo Cruz 2006 101 617 623 10.1590/S0074-02762006000600007 17072473

[B16] Joy DA Gonzalez-Ceron L Carlton JM Gueye A Fay M McCutchan TF Su XZ Local adaptation and vector-mediated population structure in *Plasmodium vivax *malaria Mol Biol Evol 2008 25 1245 1252 10.1093/molbev/msn073 18385220 PMC2386084

[B17] Miller-Butterworth CM Jacobs DS Harley EH Strong population substructure is correlated with morphology and ecology in a migratory bat Nature 2003 424 187 191 10.1038/nature01742 12853955

[B18] O'Loughlin SM Somboon P Walton C High levels of population structure caused by habitat islands in the malarial vector *Anopheles scanloni* Heredity 2007 99 31 40 10.1038/sj.hdy.6800959 17426732

[B19] O'Loughlin SM Okabayashi T Honda M Kitazoe Y Kishino H Somboon P Sochantha T Nambanya S Saikia PK Dev V Walton C Complex population history of two *Anopheles dirus *mosquito species in Southeast Asia suggests the influence of Pleistocene climate change rather than human-mediated effects J Evol Biol 2008 21 1555 1569 10.1111/j.1420-9101.2008.01606.x 18800997

[B20] Renaud S Damb JV Influence of biotic and abiotic environment on dental size and shape evolution in a Late Miocene lineage of murine rodents (Teruel Basin, Spain) Palaeogeogr Palaeoclimatol Palaeoecol 2002 184 163 175 10.1016/S0031-0182(02)00255-9

[B21] Sheldon PR Plus ça change- a model for stasis and evolution in different environments Palaeogeogr Palaeoclimatol Palaeoecol 1996 127 209 222 10.1016/S0031-0182(96)00096-X

[B22] Zarza E Reynoso VH Emerson BC Diversification in the northern neotropics: mitochondrial and nuclear DNA phylogeography of the iguana *Ctenosaura pectinata *and related species Mol Ecol 2008 17 3259 3275 10.1111/j.1365-294X.2008.03826.x 18564087

[B23] Mirabello L Conn JE Molecular population genetics of the malaria vector *Anopheles darlingi *in Central and South America Heredity 2006 96 311 321 10.1038/sj.hdy.6800805 16508661

[B24] Scarpassa VM Conn JE Population genetic structure of the major malaria vector *Anopheles darlingi *(Diptera: Culicidae) from the Brazilian Amazon, using microsatellite markers Mem Inst Oswaldo Cruz 2007 102 319 327 10.1590/S0074-02762007005000045 17568937

[B25] Conn JE Vineis JH Bollback JP Onyabe DY Wilkerson RC Povoa MM Population structure of the malaria vector *Anopheles darlingi *in a malaria-endemic region of eastern Amazonian Brazil Am J Trop Med Hyg 2006 74 798 806 16687683

[B26] Pedro PM Sallum MAM Spatial expansion and population structure of the neotropical malaria vector, *Anopheles darlingi *(Diptera: Culicidae) Biol J Linn Soc Lond 2009 97 854 866 10.1111/j.1095-8312.2009.01226.x

[B27] De Merida AM De Mata MP Molina E Porter CH Black WCI Variation in ribosomal DNA intergenic spacers among populations of *Anopheles albimanus *in South and Central America Am J Trop Med Hyg 1995 53 469 477 7485704 10.4269/ajtmh.1995.53.469

[B28] De Merida AM Palmieri M Yurrita M Molina A Molina E Black WCI Mitochondrial DNA variation among *Anopheles albimanus *populations Am J Trop Med Hyg 1999 61 230 239 10463672 10.4269/ajtmh.1999.61.230

[B29] Molina-Cruz A de Merida AM Mills K Rodriguez F Schoua C Yurrita MM Molina E Palmieri M Black WCI Gene flow among *Anopheles albimanus *populations in Central America, South America, and the Caribbean assessed by microsatellites and mitochondrial DNA Am J Trop Med Hyg 2004 71 350 359 15381818

[B30] Narang SK Seawright JA Suarez MF Genetic structure of natural populations of *Anopheles albimanus *in Colombia Journal of the American Mosquito Control Association 1991 7 437 445 1791454

[B31] Matthews SD Meehan LJ Onyabe DY Vineis J Nock I Ndams I Conn JE Evidence for late Pleistocene population expansion of the malarial mosquitoes, *Anopheles arabiensis *and *Anopheles gambiae *in Nigeria Med Vet Entomol 2007 21 358 369 18092974 10.1111/j.1365-2915.2007.00703.x

[B32] Zink RM Barrowclough GF Mitochondrial DNA under siege in avian phylogeography Mol Ecol 2008 17 2107 2121 10.1111/j.1365-294X.2008.03737.x 18397219

[B33] Dusfour I Michaux JR Harbach RE Manguin S Speciation and phylogeography of the Southeast Asian *Anopheles sundaicus *complex Infect Genet Evol 2007 7 484 493 10.1016/j.meegid.2007.02.003 17350896

[B34] Mirabello L Vineis JH Yanoviak SP Scarpassa VM Povoa MM Padilla N Achee NL Conn JE Microsatellite data suggest significant population structure and differentiation within the malaria vector *Anopheles darlingi *in Central and South America BMC Ecol 2008 8 3 10.1186/1472-6785-8-3 18366795 PMC2292152

[B35] Moreno M Salgueiro P Vicente JL Cano J Berzosa PJ de Lucio A Simard F Caccone A Do Rosario VE Pinto J Benito A Genetic population structure of *Anopheles gambiae *in Equatorial Guinea Malar J 2007 6 137 10.1186/1475-2875-6-137 17937805 PMC2100067

[B36] Antonio-Nkondjio C Ndo C Kengne P Mukwaya L Awono-Ambene P Fontenille D Simard F Population structure of the malaria vector *Anopheles moucheti *in the equatorial forest region of Africa Malar J 2008 7 120 10.1186/1475-2875-7-120 18601716 PMC2483286

[B37] Collins FH Kamau L Ranson HA Vulule JM Molecular entomology and prospects for malaria control Bull World Health Organ 2000 78 1412 1423 11196488 PMC2560650

[B38] Catteruccia F Nolan T Loukeris TG Blass C Savakis C Kafatos FC Crisanti A Stable germline transformation of the malaria mosquito *Anopheles stephensi* Nature 2000 405 959 962 10.1038/35016096 10879538

[B39] De Queiroz K Species concepts and species delimitation Syst Biol 2007 56 879 886 10.1080/10635150701701083 18027281

[B40] Cienfuegos AV Gómez GF Córdoba LA Luckhart Shirley Conn JE Correa MM Diseño y evaluación de metodologías basadas en PCR-RFLP de ITS2 para la identificación molecular de mosquitos *Anopheles *spp. (Diptera: Culicidae) de la Costa Pacífica de Colombia Rev Biomed 2008 19 35 44

[B41] Zapata MA Cienfuegos AV Quiros OI Quinones ML Luckhart S Correa MM Discrimination of seven *Anopheles *species from San Pedro de Uraba, Antioquia, Colombia, by polymerase chain reaction-restriction fragment length polymorphism analysis of its sequences Am J Trop Med Hyg 2007 77 67 72 17620632

[B42] Birungi J Munstermann L Genetic structure of *Aedes albopictus *(Diptera: Culicidae) populations based on mitochondrial ND5 sequences: evidence for an independent invasion into Brazil and United States Ann Entomol Soc Am 2002 95 125 132 10.1603/0013-8746(2002)095[0125:GSOAAD]2.0.CO;2

[B43] Lunt DH Zhang DX Szymura JM Hewitt GM The insect cytochrome oxidase I gene: evolutionary patterns and conserved primers for phylogenetic studies Insect Mol Biol 1996 5 153 165 10.1111/j.1365-2583.1996.tb00049.x 8799733

[B44] Ewing B Green P Base-Calling of Automated Sequencer Traces Using Phred. II. Error Probabilities Genome Res 1998 8 186 194 9521922

[B45] Richterich P Estimation of Errors in "Raw" DNA Sequences: A Validation Study Genome Res 1998 8 251 259 9521928 10.1101/gr.8.3.251 PMC310698

[B46] Chromas Lite^©^ 1998 Technelysium Pty Ltd, Tewantin QLD, Australia http://www.technelysium.com.au

[B47] Hall TA BioEdit: a user-friendly biological sequence alignment editor and analysis program for Windows 95/98/NT Nucleic Acids Symp Ser 1999 41 95 98

[B48] Thompson JD Gibson TJ Plewniak F Jeanmougin F Higgins DG The ClustalX windows interface: flexible strategies for multiple sequence alignment aided by quality analysis tools Nucleic Acids Res 1997 25 4876 4882 10.1093/nar/25.24.4876 9396791 PMC147148

[B49] Xia X Xie Z DAMBE: Data analysis in molecular biology and evolution J Hered 2001 92 371 373 10.1093/jhered/92.4.371 11535656

[B50] Rozas J Sanchez-DelBarrio JC Messeguer X Rozas R DnaSP, DNA polymorphism analyses by the coalescent and other methods Bioinformatics 2003 19 2496 2497 10.1093/bioinformatics/btg359 14668244

[B51] Excoffier L Laval G Schneider S Arlequin ver. 3.0: An integrated software package for population genetics data analysis Evol Bioinform Online 2005 1 47 50 19325852 PMC2658868

[B52] Tamura K Dudley J Nei M Kumar S MEGA4: Molecular Evolutionary Genetics Analysis (MEGA) software version 4.0 Mol Biol Evol 2007 24 1596 1599 10.1093/molbev/msm092 17488738

[B53] Posada D ModelTest Server: a web-based tool for the statistical selection of models of nucleotide substitution online Nucleic Acids Res 2006 34 700 703 10.1093/nar/gkl042 PMC1538795 16845102

[B54] Posada D Crandall KA Modeltest: testing the model of DNA substitution Bioinformatics 1998 14 817 818 10.1093/bioinformatics/14.9.817 9918953

[B55] Van Oosterhout C Hutchinson W Wills D Shipley P MICRO-CHECKER: software for identifying and correcting genotyping errors in microsatellite data Mol Ecol Notes 2004 4 535 538 10.1111/j.1471-8286.2004.00684.x

[B56] Peakall R Smouse PE GENALEX 6: Genetic Analysis in Excel. Population genetic software for teaching and research Mol Ecol Notes 2006 6 288 295 10.1111/j.1471-8286.2005.01155.x PMC3463245 22820204

[B57] Goudet J FSTAT version 2.9.3.2. A computer software to calculate F-statistics J Hered 1995 86 485 486

[B58] Rousset F Genepop'007: a complete reimplementation of the Genepop software for Windows and Linux Mol Ecol Resour 2008 8 103 106 10.1111/j.1471-8286.2007.01931.x 21585727

[B59] Holm S A simple sequentially rejective multiple test procedure Scand J Stat 1979 6 65 70

[B60] Weir B Cockerham C Estimating F-statistics for the analysis of population structure Evolution 1984 38 1358 1370 10.2307/2408641 28563791

[B61] Peel D Ovenden J Peel S NeEstimator: software for estimating effective population size, Versión 1.3 Queensland Government, Department of Primary Industries and Fisheries, Brisbane, Queensland 2004

[B62] Google Earth 4.3 2008 Europa-Technologies http://earth.google.com/

[B63] Dupanloup I Schneider S Excoffier L A simulated annealing approach to define the genetic structure of populations Mol Ecol 2002 11 2571 2581 10.1046/j.1365-294X.2002.01650.x 12453240

[B64] Piry S Alapetite A Cornuet JM Paetkau D Baudouin L Estoup A GeneClass2: A Software for Genetic Assignment and First-Generation Migrant Detection J Hered 2004 95 536 539 10.1093/jhered/esh074 15475402

[B65] Rannala B Mountain JL Detecting immigration by using multilocus genotypes Proc Natl Acad Sci USA 1997 94 9197 9201 10.1073/pnas.94.17.9197 9256459 PMC23111

[B66] Corander J Marttinen P Siren J Tang J Enhanced Bayesian modelling in BAPS software for learning genetic structures of populations BMC bioinformatics 2008 9 539 10.1186/1471-2105-9-539 19087322 PMC2629778

[B67] Miller M A Windows program for the analysis of allozyme and molecular population genetic data (TFPGA) 1997 Department of Biological Sciences, Northern Arizona University, Flagstaff, USA

[B68] Mantel N The detection of disease clustering and a generalized regression approach Cancer Res 1967 27 209 220 6018555

[B69] Jensen JL Bohonak AJ Kelley ST Isolation by distance, web service, v.3.15 BMC Genet 2005 6 13 10.1186/1471-2156-6-13 15760479 PMC1079815

[B70] Kimura M The neutral theory of Molecular Evolution 1983 Cambridge, Massachusetts: Cambridge University Press

[B71] Tajima F Statistical method for testing the neutral mutation hypothesis by DNA polymorphism Genetics 1989 123 585 595 2513255 10.1093/genetics/123.3.585 PMC1203831

[B72] Fu YX Statistical tests of neutrality of mutations against population growth, hitchhiking and background selection Genetics 1997 147 915 925 9335623 10.1093/genetics/147.2.915 PMC1208208

[B73] Harpending H Signature of ancient population growth in a low-resolution mitochondrial DNA mismatch distribution Hum Biol 1994 66 591 600 8088750

[B74] Rogers AR Harpending H Population growth makes waves in the distribution of pairwise genetic differences Mol Biol Evol 1992 9 552 569 1316531 10.1093/oxfordjournals.molbev.a040727

[B75] Slatkin M Hudson RR Pairwise comparisons of mitochondrial DNA sequences in stable and exponentially growing populations Genetics 1991 129 555 562 1743491 10.1093/genetics/129.2.555 PMC1204643

[B76] Clement M Posada D Crandall KA TCS: a computer program to estimate gene genealogies Mol Ecol 2000 9 1657 1660 10.1046/j.1365-294x.2000.01020.x 11050560

[B77] Crandall KA Templeton AR Empirical tests of some predictions from coalescent theory with applications to intraspecific phylogeny reconstruction Genetics 1993 134 959 969 8349118 10.1093/genetics/134.3.959 PMC1205530

[B78] Posada D Crandall KA Intraspecific gene genealogies: trees grafting into networks Trends Ecol Evol 2001 16 37 45 10.1016/S0169-5347(00)02026-7 11146143

[B79] Uthicke S Benzie JAH Gene flow and population history in high dispersal marine invertebrates: mitochondrial DNA analysis of *Holothuria nobilis *(Echinodermata: Holothuroidea) populations from the Indo-Pacific Mol Ecol 2003 12 2635 2648 10.1046/j.1365-294X.2003.01954.x 12969467

[B80] Huson DH Bryant D Aplication of phylogenetic networks in Evolutionary studies Mol Biol Evol 2006 23 254 267 10.1093/molbev/msj030 16221896

[B81] Swofford DL PAUP* version 4.0. Phylogenetic Analysis Using Parsimony (and Other Methods) 2000 Sinauer Associates, Inc, Sunderland, Massachusetts, USA

[B82] Sallum MAM Schultz TR Foster PG Aronstein K RA W RC W Phylogeny of *Anophelinae *(Diptera: Culicidae) based on nuclear ribosomal and mitochondrial DNA sequences Syst Entomol 2002 27 361 382 10.1046/j.1365-3113.2002.00182.x

[B83] Cornuet JM Luikart G Description and power analysis of two tests for detecting recent population bottlenecks from allele frequency data Genetics 1996 144 2001 2014 8978083 10.1093/genetics/144.4.2001 PMC1207747

[B84] Beard CB Hamm DM Collins FH The mitochondrial genome of the mosquito *Anopheles gambiae *: DNA sequence, genome organization, and comparisons with mitochondrial sequences of other insects Insect Mol Biol 1993 2 103 124 10.1111/j.1365-2583.1993.tb00131.x 9087549

[B85] Tamura K Nei M Estimation of the number of nucleotide substitutions in the control region of mitochondrial DNA in humans and chimpanzees Mol Biol Evol 1993 10 512 526 8336541 10.1093/oxfordjournals.molbev.a040023

[B86] Tavaré S Miura RM Some probabilistic and statistical problems in the analysis of DNA sequences. Some mathematical questions in biology - DNA sequence analysis 1986 Providence, RI. Amer Math Soc

[B87] Castelloe J Templeton AR Root probabilities for intraspecific gene trees under neutral coalescent theory Molecular phylogenetics and evolution 1994 3 102 113 10.1006/mpev.1994.1013 8075830

[B88] Powell JR Caccone A Amato GD Yoon C Rates of nucleotide substitution in *Drosophila *mitochondrial DNA and nuclear DNA are similar Proc Natl Acad Sci USA 1986 83 9090 9093 10.1073/pnas.83.23.9090 3097641 PMC387080

[B89] Walton C Handley JM Tun-Lin W Collins FH Harbach RE Baimai V Butlin RK Population structure and population history of *Anopheles dirus *mosquitoes in Southeast Asia Mol Biol Evol 2000 17 962 974 10833203 10.1093/oxfordjournals.molbev.a026377

[B90] Behura SK Molecular marker systems in insects: current trends and future avenues Mol Ecol 2006 15 3087 3113 10.1111/j.1365-294X.2006.03014.x 16968257

[B91] Walton C Thelwell NJ Priestman A Butlin RK The use of microsatellites to study gene flow in natural populations of *Anopheles *malaria vectors in Africa: potential and pitfalls J Am Mosq Control Assoc 1998 14 266 272 9813823

[B92] Keppler WJ Kitzmiller JB Rabbani MG The salivary gland chromosomes of *Anopheles albimanus* Mosq News 1973 33 42 49

[B93] Onyabe DY Conn JE Genetic differentiation of the malaria vector *Anopheles gambiae *across Nigeria suggests that selection limits gene flow Heredity 2001 87 647 658 10.1046/j.1365-2540.2001.00957.x 11903560

[B94] Walton C Handley JM Collins FH Baimai V Harbach RE Deesin V RK B Genetic population structure and introgression in *Anopheles dirus *B: mosquitoes in South-east Asia Mol Ecol 2001 10 569 580 10.1046/j.1365-294x.2001.01201.x 11298969

[B95] Callen DF Thompson AD Shen Y Phillips HA Richards RI Mulley JC Sutherland GR Incidence and origin of "null" alleles in the (AC)n microsatellite markers Am J Hum Genet 1993 52 922 927 8488841 PMC1682051

[B96] McCartney M Brayer K Levitan D Polymorphic microsatellite loci from the red sea urchin, *Strongylocentrotus franciscanus*, with comments on heterozygote deficit Mol Ecol Notes 2004 4 226 228 10.1111/j.1471-8286.2004.00624.x

[B97] Hartl DL Clark AG Principles of population genetics 2007 4 Sunderland, MA, USA: Sinauer Associates, Inc

[B98] Balloux F Goudet J Statistical properties of population differentiation estimators under stepwise mutation in a finite island model Mol Ecol 2002 11 771 783 10.1046/j.1365-294X.2002.01474.x 11972763

[B99] Donnelly MJ Simard F Lehmann T Evolutionary studies of malaria vectors Trends Parasitol 2002 18 75 80 10.1016/S1471-4922(01)02198-5 11832298

[B100] Posso CE Gonzalez R Cardenas H Gallego G Duque MC Suarez MF Random amplified polymorphic DNA analysis of *Anopheles nuneztovari *(Diptera: Culicidae) from Western and northeastern Colombia Mem Inst Oswaldo Cruz 2003 98 469 476 12937756 10.1590/s0074-02762003000400007

[B101] Gonzalez R Wilkerson R Suarez MF Garcia F Gallego G Cardenas H Posso CE Duque MC A population genetics study of *Anopheles darlingi *(Diptera: Culicidae) from Colombia based on random amplified polymorphic DNA-polymerase chain reaction and amplified fragment lenght polymorphism markers Mem Inst Oswaldo Cruz 2007 102 255 262 10.1590/S0074-02762007005000037 17568929

[B102] Dantur Juri MJ Zaidenberg M Claps GL Santana M Almiron WR Malaria transmission in two localities in north-western Argentina Malar J 2009 8 18 10.1186/1475-2875-8-18 19152707 PMC2644309

[B103] Coluzzi M Petrarca V Di Deco MA Chromosomal inversion intergradation and incipient speciation in *Anopheles gambiae* Boll Zool 1985 52 45 63

[B104] Karch S Dellile MF Guillet P Mouchet J African malaria vectors in European aircraft Lancet 2001 357 235 10.1016/S0140-6736(05)71339-8 11213131

[B105] Weir BS Hill WG Estimating F-statistics Annu Rev Genet 2002 36 721 750 10.1146/annurev.genet.36.050802.093940 12359738

[B106] Donnelly MJ Licht MC Lehmann T Evidence for recent population expansion in the evolutionary history of the malaria vectors *Anopheles arabiensis *and *Anopheles gambiae* Mol Biol Evol 2001 18 1353 1364 11420373 10.1093/oxfordjournals.molbev.a003919

[B107] Lehmann T Hawley WA Grebert H Collins FH The effective population size of *Anopheles gambiae *in Kenya: implications for population structure Mol Biol Evol 1998 15 264 276 9501493 10.1093/oxfordjournals.molbev.a025923

[B108] Donnelly MJ Cuamba N Charlwood JD Collins FH Townson H Population structure in the malaria vector, *Anopheles arabiensis *patton, in East Africa Heredity 1999 83 408 417 10.1038/sj.hdy.6885930 10583542

[B109] Kayondo JK Mukwaya LG Stump A Michel AP Coulibaly MB Besansky NJ Collins FH Genetic structure of *Anopheles gambiae *populations on islands in northwestern Lake Victoria, Uganda Malar J 2005 4 59 10.1186/1475-2875-4-59 16336684 PMC1327676

[B110] Lehmann T Blackston CR Besansky NJ Escalante AA Collins FH Hawley WA The Rift Valley complex as a barrier to gene flow for *Anopheles gambiae *in Kenya: the mtDNA perspective J Hered 2000 91 165 168 10.1093/jhered/91.2.165 10768135

[B111] Lehmann T Hawley WA Grebert H Danga M Atieli F Collins FH The Rift Valley complex as a barrier to gene flow for *Anopheles gambiae *in Kenya J Hered 1999 90 613 621 10.1093/jhered/90.6.613 10589511

[B112] Olano V Carrasquilla G Mendez F Transmission of urban malaria in Buenaventrua, Colombia: entomological features Rev Panam Salud Publica 1997 1 287 294 10.1590/S1020-49891997000400005 9303813

[B113] Poveda G Rojas W Quiñones M Vélez I Mantilla R Ruiz D Zuluaga J Rua G Coupling between Annual and ENSO Timescales in the Malaria-Climate Association in Colombia Environ Health Perspect 2001 109 489 493 10.2307/3454707 11401760 PMC1240308

