# Supplementary material for: Population structure analyses and demographic history of the malaria vector Anopheles albimanus from the Caribbean and the Pacific regions of Colombia
Source: Malar J. 2009 Nov 19;8:259. doi: 10.1186/1475-2875-8-259 (PMC2789746; doi:10.1186/1475-2875-8-259)
Supplement: Additional file 1 — Estimates of effective population size (Ne) and heterozygosity tests based on MS data for An. albimanus. The table provided include the estimated values of effective population size (Ne) and heterozygosity tests based on MS data for An. albimanus from eight sites from the Caribbean and the Pacific regions of Colombia. [file 1475-2875-8-259-S1.DOC]

**Additional file 1: Estimates of effective population size (*Ne*) and heterozygosity tests based on MS data in *An. albimanus***

|  |  |  | **Populations** | | | | | | | |
| --- | --- | --- | --- | --- | --- | --- | --- | --- | --- | --- |
|  | **Model** |  | **ACH** | **SRL** | **MON** | **TUR** | **NUQ** | **BUE** | **STU** | **MTU** |
| ***Ne*** | LD |  | 238.9 | 375.3 | 230.9 | ∞ | ∞ | 384.2 | ∞ | ∞ |
|  | CI |  | 56-∞ | 59.9-∞ | 61.3-∞ | 97.2-∞ | 108.9-∞ | 77.5-∞ | 41.2-∞ | 38.5-∞ |
|  | HE |  | ∞ | ∞ | ∞ | ∞ | ∞ | ∞ | ∞ | ∞ |
|  | CI |  | NA | NA | NA | NA | NA | NA | NA | NA |
| Heterozygosity tests | **IAM** | *H_e_* < *H_eq_* | 0 | 0 | 1 | 0 | 0 | 0 | 1 | 0 |
|  |  | *H_e_* > *H_eq_* | 4 | 4 | 3 | 4 | 4 | 4 | 3 | 4 |
|  |  | P (*H_e_* > *H_eq_*) | 0.11839 | 0.12530 | 0.42193 | 0.12351 | 0.13037 | 0.13217 | 0.46481 | 0.12065 |
|  | **SMM** | *H_e_* < *H_eq_* | 3 | 2 | 2 | 2 | 4 | 3 | 4 | 2 |
|  |  | *H_e_* > *H_eq_* | 1 | 2 | 2 | 2 | 0 | 1 | 0 | 2 |
|  |  | P (*H_e_* > *H_eq_*) | 0.18788 | 0.54758 | 0.53646 | 0.55269 | 0.45583 | 0.19309 | 0.02769 | 0.53993 |
|  | **TPM** | *H_e_* < *H_eq_* | 2 | 1 | 1 | 2 | 1 | 2 | 3 | 2 |
|  |  | *H_e_* > *H_eq_* | 2 | 3 | 3 | 2 | 3 | 2 | 1 | 2 |
|  |  | P (*H_e_* > *H_eq_*) | 0.53129 | 0.46098 | 0.45712 | 0.54588 | 0.02996 | 0.54930 | 0.18641 | 0.53128 |

LD: Linkage disequilibrium model, HE: Heterozygote Excess model, CI: Confidence intervals, *He*>*Heq*: number of loci showing Heterozygote Excess, P (*He*>*Heq*): *P-value* of sign tests (*p*<0.05) to verify the significance of the number of loci in which *He*>*Heq*. SMM: Stepwise Mutation Model, IAM: Infinite Alleles Model and TPM: Two Phase Model.
