# Supplementary material for: Population structure analyses and demographic history of the malaria vector Anopheles albimanus from the Caribbean and the Pacific regions of Colombia
Source: Malar J. 2009 Nov 19;8:259. doi: 10.1186/1475-2875-8-259 (PMC2789746; doi:10.1186/1475-2875-8-259)
Supplement: Additional file 2 — Results of neutrality tests based on COI sequences of An. albimanus from Colombia. The table provided describe the estimated values of neutrality tests based on COI sequences of An. albimanus from eight sites from the Caribbean and the Pacific regions of Colombia. [file 1475-2875-8-259-S2.DOC]

**Additional file 2: Results of neutrality tests based on *COI* sequences of *An. albimanus* from Colombia**

| **Population** |  | **Tajima’s *D*** | **Fu’s *F_s_*** |
| --- | --- | --- | --- |
| Caribbean Region |  | -2.08901^**^ | -25.9802^**^ |
| ACH |  | -1.41920 | -5.608^**^ |
| SRL |  | -1.78035^**^ | -12.345^**^ |
| MON |  | -1.92139^**^ | -15.114^**^ |
| TUR |  | -1.56006^*^ | -16.548^**^ |
| Pacific Region |  | -0.78210 | -21.8806^**^ |
| NUQ |  | -0.52831 | 0.149 |
| PIZ |  | -0.31630 | -0.530 |
| BUE |  | -0.45391 | -0.469 |
| TUM |  | -0.24583 | -6.911^**^ |
| All localities |  | -0.31859 | -67.001 |

^**^*p*<0.02; ^*^ *p*<0.05
